# Cellular basis of omentum activation and expansion revealed by single-cell RNA sequencing using a parabiosis model

**DOI:** 10.1038/s41598-021-93330-5

**Published:** 2021-07-06

**Authors:** Kazuhiko Ishigaki, Keiki Kumano, Kyohei Fujita, Hiroo Ueno

**Affiliations:** Department of Stem Cell Pathology, Kansai Medical University, 2-5-1 Shin-machi, Hirakata, Osaka 573-1010 Japan

**Keywords:** Regeneration, Cell growth

## Abstract

Although the physiological function of the omentum remains elusive, it has been proposed that it plays an important role in fat storage, immune regulation, and regeneration of injured tissues and organs. Although the omentum undergoes expansion upon activation, reports on the accurate assessment of increased cell types and the origin of the increased cells remain limited. To investigate this aspect, the omenta of parabiotic mice were subjected to activation using distinct fluorescent markers and single-cell RNA sequencing (scRNA-seq) was performed to identify circulation-derived omental cells. We found that a considerable number of circulating cells contributed to the activation of the omentum. The omental cells derived from circulating cells exhibited morphological features similar to those of fibroblasts. scRNA-seq revealed the existence of a novel cell population that co-expressed macrophage and fibroblast markers in the activated omentum, suggesting that it corresponded to circulating macrophage-derived fibroblast-like cells. Lineage tracing experiments revealed that most fibroblasts in the activated omentum were not derived from WT1-positive mesenchymal progenitors. The cell cluster also expressed various chemokine genes, indicating its role in the activation and recruitment of immune cells. These results provide important information regarding the activation of the omentum.

The omentum is a flat tissue mainly composed of adipocytes, fibroblasts, and immune cells, and exists on the surface of intraperitoneal organs. It has been proposed that the omentum plays an important role in fat storage, immune regulation, and tissue regeneration^1,2^. The omentum is activated and increases in size during acute inflammation^1,3^. Although this phenomenon is considered to exert protective effects on tissues in conditions such as acute peritonitis and intraperitoneal organ injury, the underlying mechanisms remain unknown. However, these protective effects of the omentum have already been considered in regenerative surgery^2^. Therefore, a deeper understanding of the activation of omentum during inflammation is important for the development of regenerative medicine-based strategies.

To study omentum activation in mice, an intraperitoneal injection of polyacrylamide beads was performed to induce acute inflammation similar to that reported in previous studies^4^. Fluorescence-activated cell sorting (FACS) analysis was performed on omental cells of polyacrylamide bead-injected mice, and the proportion of at least two groups of cells, namely myeloid-derived immune cells and cells indistinguishable from mesenchymal stem cells (MSCs), was found to increase in the activated omenta^4^. However, it has not been investigated whether the cells that increase in proportion in the activated omentum are derived from the omentum itself or from cells in circulation. The types of cells that increase in proportion remain unexamined.

The fate of circulating cells has been frequently analyzed using parabiosis, in which the skin and body cavities of two mice are surgically connected^5^. In parabiotic mice, blood vessels are connected, and circulating cells move freely back and forth between the two mice. In the case of parabiosis between green fluorescent protein (GFP)-positive and GFP-negative mice, the GFP-positive cells in the tissues of GFP-negative mice were derived from circulating cells. However, it is difficult to accurately exclude the possibility that cells are generated by cell fusion between donor-derived cells and recipient-derived cells. To exclude fused cells from the analyses, we utilized *Rosa26*^*EGFP/*+^ and *Rosa26*^*ECFP/*+^ knock-in mice^6^, in which fused cells were identified as cells co-expressing EGFP and ECFP.

Furthermore, scRNA-seq is one of the most powerful methods used to accurately assess the composition of the cell population of interest at the single-cell level, and various algorithms have been developed for different experimental purposes^7^.

In this study, parabiosis of mice with two distinct fluorescent markers was performed, and donor-derived omental cells in the recipient omentum were purified and subjected to scRNA-seq.

## Results

### Histological analysis of enlarged omentum in mice

**Figure 1:**
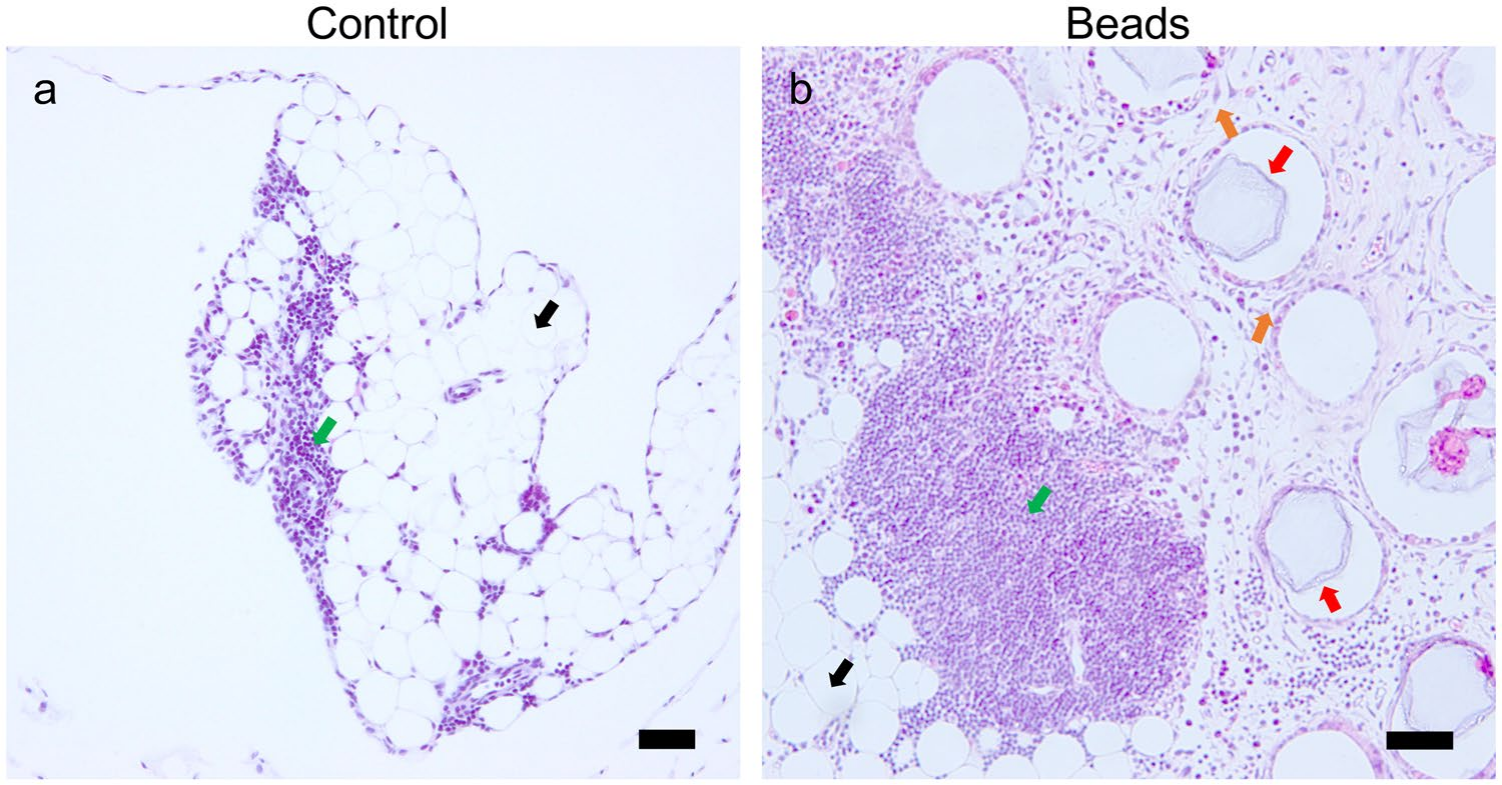
Representative images of the omenta of control and bead-injected mice Immunohistochemistry of the omenta of C57BL/6J mice subjected to H&E staining analysis. (**a)** Image of the tissue derived from the control mouse. (**b)** Image of the tissue derived from the bead-injected mouse. Bead-based treatment was conducted for a period of 7 days. Black arrows (**a** and **b**) indicate adipocytes. Red arrows (**b**) indicate polyacrylamide beads. Green arrows (**a** and **b**) indicate milky spots. Orange arrows (**b**) indicate fibroblasts. Scale bars = 50 µm.

Intraperitoneal injection of polyacrylamide beads reportedly activates and expands the omentum^3,4^, and macrophages predominantly increase in number^3^. Therefore, we injected polyacrylamide beads into the peritoneal cavity of mice, collected the omentum 7 days after injection, and analyzed the enlarged omentum histologically. In the steady state, the omentum consisted of adipocyte-rich regions and exhibited several milky-white spots primarily comprising lymphocytes and residual macrophages^1^ (Fig. 1a). We observed an increase in the size of the omentum and the number of cells (Fig. 1b). The beads were encapsulated by collagen fibers (Supplementary Fig. 1d–f), whereas the omentum of mice not injected with beads presented with few collagen fibers (Supplementary Fig. 1a–c). The non-fat region underwent expansion after activation via bead injection, and the proportion of CD45+ hematopoietic cells and ER-TR7+ fibroblasts increased remarkably (Supplementary Fig. 2). The hematopoietic cells showing the highest increase in number were identified as F4/80 (+) macrophages, which accumulated around the beads (Supplementary Fig. 3), consistent with the results reported by a previous study^3^.

### Adipocyte progenitor cells minimally contribute to the increased number of fibroblasts

**Figure 2:**
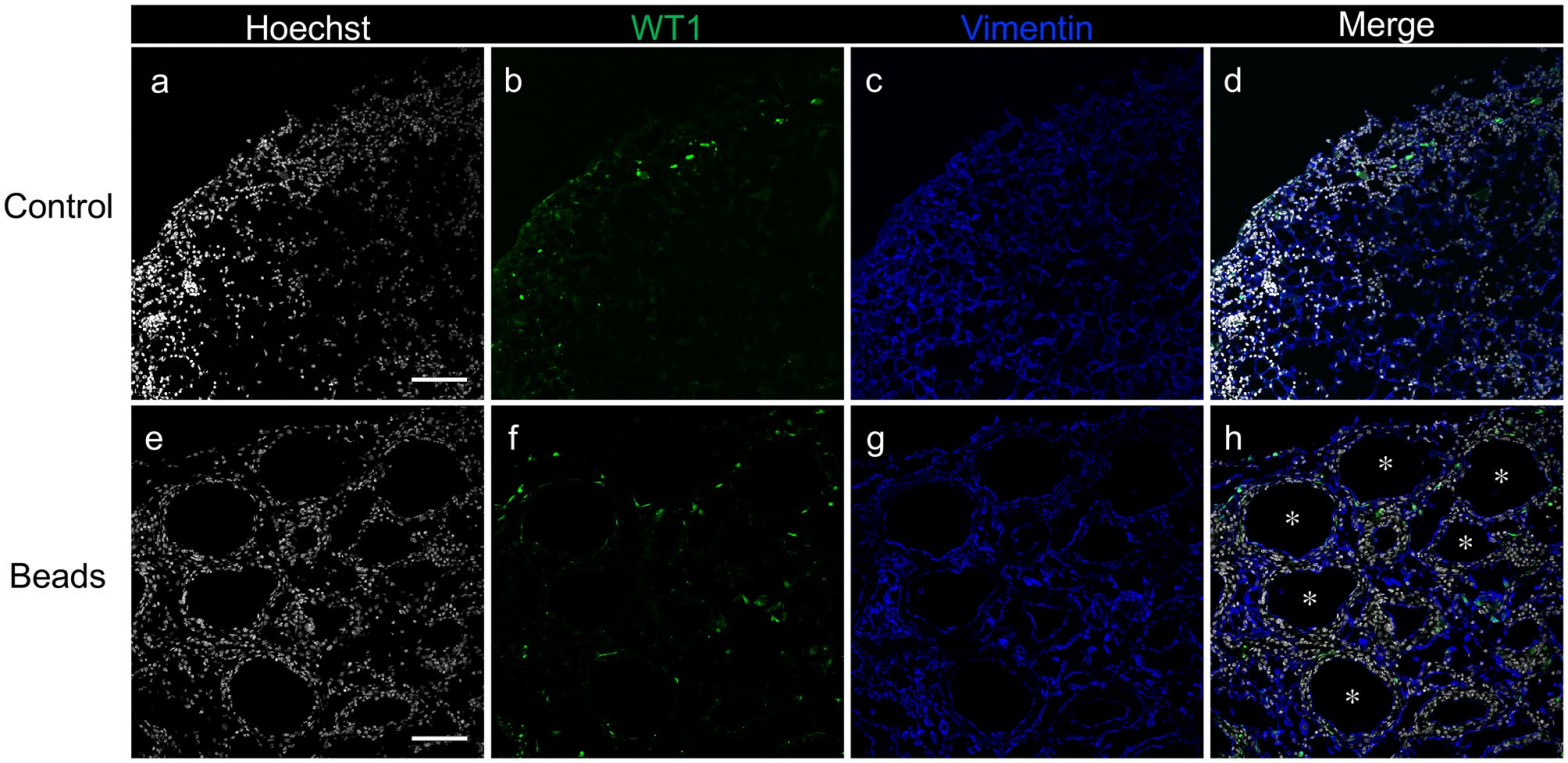
Representative confocal images of the omenta of control and bead-injected *WT1*^*CreER2T/*+^ and *Rosa26*^*loxp-stop-loxp-YFP/*+^ mice. Eight-week-old mice were intraperitoneally injected with tamoxifen and polyacrylamide beads. Mice were sacrificed 7 days after injection. Images (**a**–**d**) show the omentum of the control mouse, and (**e**–**h**) show the omentum of the bead-injected mouse. (**a** and **e)** Hoechst nuclear staining. (**b** and **f**) Expression pattern of WT1 in the same field as (**a)** and (**e)**, respectively. (**c** and **g**) Expression pattern of vimentin in the same field as (**a**) and (**e**), respectively. (**d** and **h**) Merged images of (**a**–**c**) and (**e**–**g**), respectively. Asterisks (**h**) indicate polyacrylamide beads. Scale bars = 100 µm.

The activated omentum contains various types of cells, including omnipotent stem cells that are indistinguishable from mesenchymal stem/progenitor cells^3,4^. Furthermore, WT1 expression highlights mesenchymal progenitors present in visceral fat, including the omentum^8^. To examine whether mesenchymal progenitors were the origin of fibroblasts in the activated omentum, we performed lineage tracing analysis using *WT1*^*CreERT2/*+^/*Rosa26*^*loxp-stop-loxpYFP/*+^ mice injected with polyacrylamide beads and tamoxifen (9 mg/40 g of body weight) for a period of 7 days. There were few vimentin (+) fibroblasts among the progeny of *WT1*-expressing mesenchymal progenitor cells; therefore, most fibroblasts did not originate from the WT1-positive mesenchymal progenitor cells (Fig. 2).

### Parabiosis model reveals the contribution of circulating cells to fibroblast-like cells

**Figure 3:**
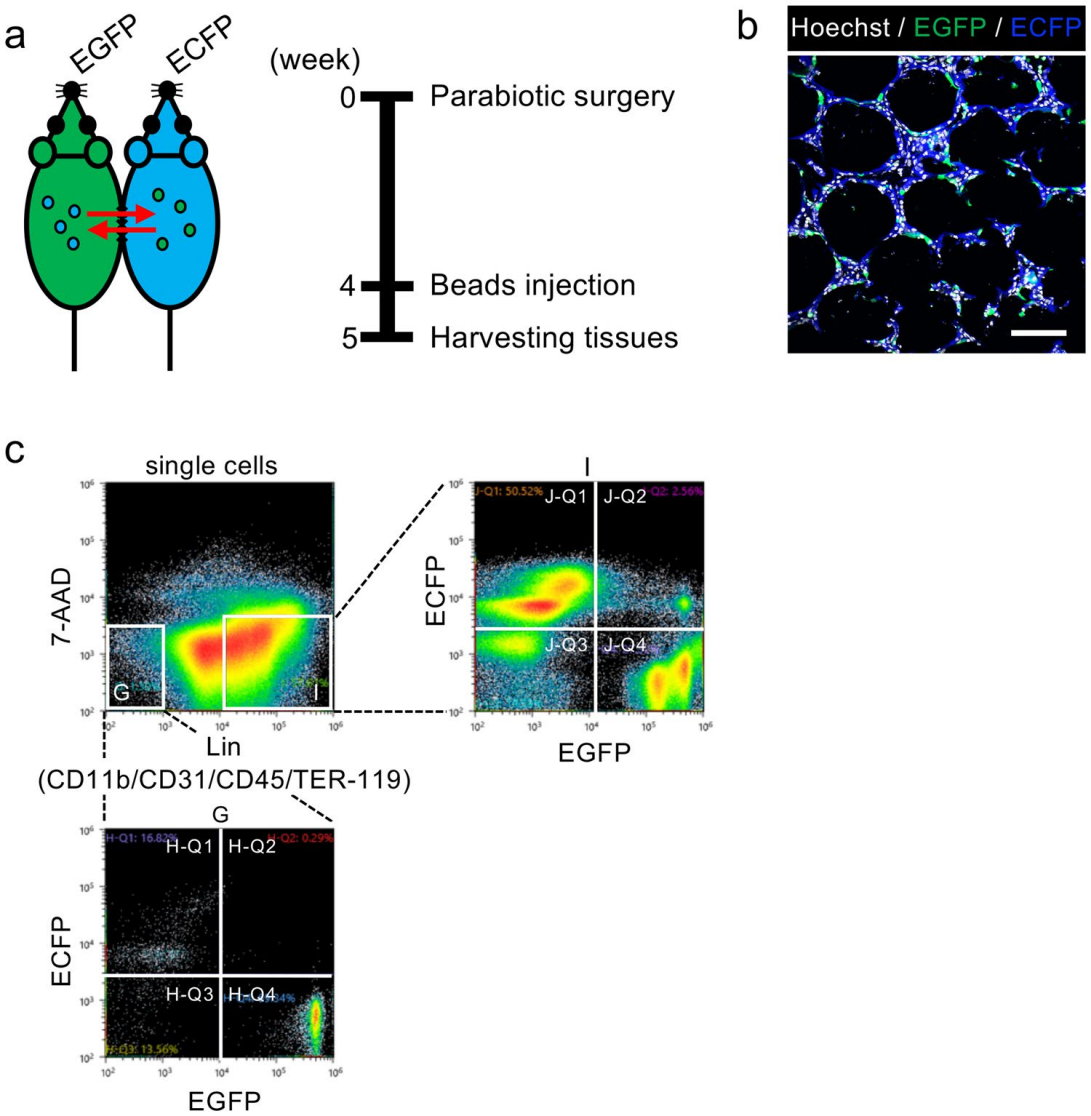
Scheme of parabiosis experiments conducted using *Rosa26*^*ECFP/*+^ and *Rosa26*^*EGFP/*+^ mice. (**a**) *Rosa26*^*ECFP/*+^ mice were paired with *Rosa26*^*EGFP/*+^ mice and injected intraperitoneally with polyacrylamide beads 4 weeks after surgery. “EGFP” and “ECFP” denote a *Rosa26*^*EGFP/*+^ and *Rosa26*^*ECFP/*+^ mice, respectively. (**b**) Representative confocal image of the omenta of parabiotic *Rosa26*^*ECFP/*+^ mice paired with *Rosa26*^*EGFP/*+^ mice. EGFP^+^ cells were derived from *Rosa26*^*EGFP/*+^ mice. (**c**) Representative flow cytometric plots of omental cells derived from *Rosa26*^*ECFP/*+^ mice paired with *Rosa26*^*EGFP/*+^ mice. Then, 7-AAD^−^/Lin^−^/ECFP^−^/EGFP^+^ cells (gate “H-Q4”) and 7-AAD^−^/Lin^+^/ECFP^−^/EGFP^+^ cells (gate “J-Q4”) were sorted for conducting scRNA-seq. Scale bar = 50 µm.

**Figure 4:**
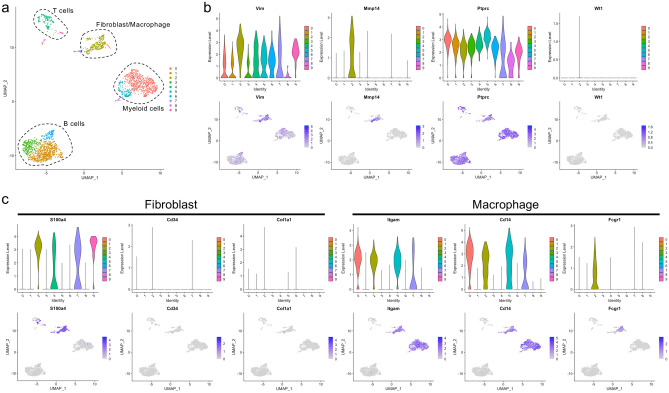
scRNA-Seq using omentum cells. (**a**) UMAP of scRNA-seq (*n* = 2456 cells) revealed 10 clusters. (**b**) Violin (top) and feature plots (bottom) illustrating fibroblast markers *Vim* and *Mmp14*, pan-hematopoietic marker *Ptprc* (also known as *CD45*), and adipose-derived stem cell marker *Wt1*. (**c**) Violin (top) and feature plots (bottom) illustrating fibroblast markers *S100a4, CD34*, and *Col1a1*, and macrophage markers *Itgam* (also known as *CD11b*), *CD14*, and *Fcgr1* (also known as *CD64*).

To examine whether circulating cells contributed to fibroblast-like cells and to exclude cell fusion events, we performed parabiosis between *Rosa26*^*EGFP/*+^ and *Rosa26*^*ECFP/*+^ mice (Fig. 3a, left). Blood flow stabilization occurred 4 weeks after subjection to parabiotic surgery, and approximately equal chimerism of EGFP and ECFP in blood cells was confirmed as per methods reported previously^9^. Polyacrylamide beads were then intraperitoneally injected into the parabiotic mice to activate the omenta. The enlarged activated omentum of a *Rosa26*^*ECFP/*+^ mouse was collected 7 days after subjection to bead injection (Fig. 3a, right). We observed a considerable number of EGFP-positive cells in the omentum, and these were morphologically similar to fibroblasts that possibly migrated from the *Rosa26*^*EGFP/*+^ mouse (Fig. 3b). FACS analysis revealed that the EGFP-positive cells in the omentum included cells other than hematopoietic and endothelial cells, as they were negative for CD45 and CD31 (Fig. 3c). Next, the migrated EGFP-positive cells from the omentum of a *Rosa26*^*ECFP/*+^ mouse were subjected to sorting and scRNA-seq analysis. The sorted cells were classified into 10 clusters (Fig. 4a). *Ptprc* (CD45) expression was observed in all clusters, which were assumed to be derived from hematopoietic cells (Fig. 4b). Clusters 4 and 9 were identified as CD3d-positive T cells, including CD4-positive helper T cells and CD8a-positive cytotoxic T cells. Clusters 1, 3, and 6 were identified as B cells expressing CD19, Ms4a1, and Cr2. Clusters 0 and 5 were identified as S100a9-, CD33-, and Csf3r-positive myeloid lineage cells (Fig. 4b, Supplementary Fig. 4a, Supplementary Table 1). Notably, Cluster 2 was found to be a cell population co-expressing fibroblast markers, including vimentin and Mmp14^10^ (Fig. 4b), and macrophage markers, namely Adgre1 (F4/80), CD68, and Lrp1 (CD91) (Fig. 4b, c, Supplementary Table 1) (Fig. 4a, Supplementary Fig. 4a, Supplementary Table 1). Gene ontology analysis revealed that the terms inflammatory response, myeloid leukocyte migration, and monocyte-related genes were strongly enriched (Supplementary Fig. 5). S100a4, also termed fibroblast-specific protein-1 (FSP-1), was previously reported as a marker for macrophage-derived fibroblast-like cells^11^. Cluster 2 expressed high S100a4 (FSP-1) levels but low levels of extracellular matrix genes (Fig. 4c). It was also confirmed that they secreted several chemokines, such as Ccl6, Ccl9, Ccl24, and Cxcl16 (Supplementary Table 1; top100 genes, Supplementary Fig. 6) and S100a4 (FSP-1), which regulate inflammation (Fig. 4C).

**Figure 5:**
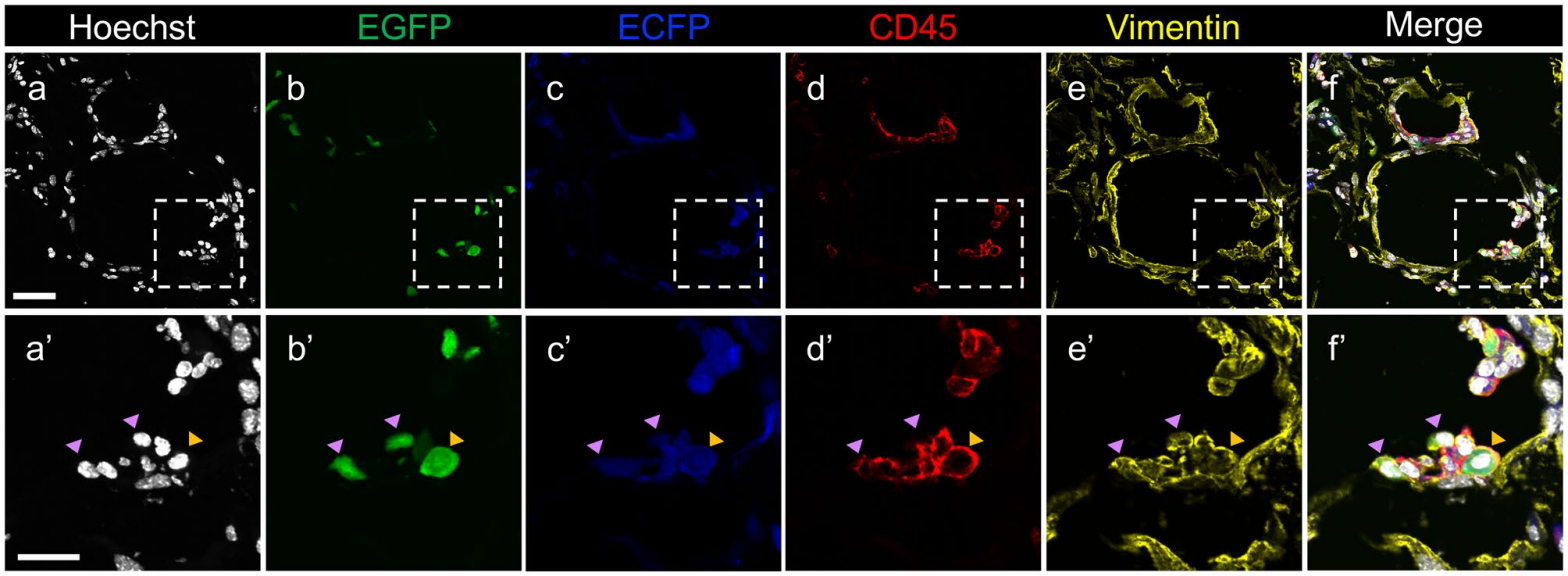
Representative confocal images of the omenta of parabiotic *Rosa26*^*ECFP/*+^ mice paired with *Rosa26*^*EGFP/*+^ mice. *Rosa26*^*ECFP/*+^ mice were paired with *Rosa26*^*EGFP/*+^ mice and injected with polyacrylamide beads 4 weeks after surgery. Mice were sacrificed 7 days after injection. Images (**a**–**f′**) show the omentum of the *Rosa26*^*ECFP/*+^ mouse. (**a**) Hoechst nuclear staining. (**b**) Expression pattern of EGFP in the same field as (**a**). (**c**) Expression pattern of ECFP in the same field as (**a**). (**d**) Expression pattern of CD45 in the same field as (**a**). (**e**) Expression pattern of vimentin in the same field as (**a**). (**f**) Merged image of (**a**–**e**). (**a′**–**f′**) shows enlarged images of dotted line squares in (**a**–**f**). Purple arrowheads indicate CD45^−^/Vimentin^+^ cells expressing EGFP. Orange arrowhead indicates CD45^+^/Vimentin^+^ cells expressing EGFP. Scale bars = 50 µm.

In contrast, CD45 expression, the exclusion criteria for mesenchymal progenitor cells, was observed in all clusters, and no population expressed mesenchymal progenitor markers such as WT1; therefore, we concluded that they were not derived from mesenchymal progenitor cells but were derived from hematopoietic cells (Fig. 4b). Histological analysis showed that the migrating vimentin (+) and EGFP (+) fibroblasts were present in the omentum of *Rosa26*^*ECFP/*+^ mice (approximately 10% vimentin (+) fibroblasts) (Fig. 5). Interestingly, contrary to the scRNA-seq results, histological analysis showed that among the migrated EGFP-positive cells, both CD45-positive and CD45-negative cells were observed around the beads (Fig. 5, Supplementary Fig. 7). The results of scRNA-seq, a highly sensitive method adopted for detecting expressed genes, showed that CD45-negative fibroblast-like cells (Fig. 5) expressed a low level of CD45. This suggested that these cells were in the process of differentiation from macrophages to fibroblast-like cells and indicated the loss of hematopoietic cell features. These results validate the previously proposed model established in other tissues, in which migrating macrophages undergo differentiation into fibroblast-like cells^11^.

## Discussion

As described above, the omentum is a fat storage site that plays important roles in immune regulation and promotes tissue regeneration^2–4^. It has been proposed that the omentum contains at least two groups of cells, namely immunomodulatory myeloid-derived suppressor cells and omnipotent stem cells, that support tissue repair and that are indistinguishable from mesenchymal stem cells^4^. This study aimed to understand the mechanism underlying omentum activation and enlargement during acute inflammation, which are key aspects for investigating its various functions. Injection of polyacrylamide beads into the peritoneal cavity is an established procedure for omentum activation in mice^4^. FACS analyses were performed to study omental cells^12^. However, cells originating from the omentum and those derived from circulating cells have not been distinguished using the parabiosis method. Moreover, scRNA-seq has not been performed to investigate the increased number of omental cells during inflammation. Parabiosis performed between *Rosa26*^*EGFP/*+^ and *Rosa26*^*ECFP/*+^ mice helped to clearly distinguish host-derived and circulation-derived omental cells and to exclude cells that underwent cell fusion.

In our study, most cells that increased in number in the omentum were morphologically similar to fibroblasts and hematopoietic cells, but were not similar to fat cells, and the migrating cells contributed to the generation of both lineages (Figs. 1, 3b, 5, Supplementary Fig. 2). However, scRNA-seq revealed that all cell clusters identified in the migrating cells were CD45-positive, suggesting that they originated from circulating hematopoietic cells. Interestingly, scRNA-seq revealed that Cluster 2 co-expressed fibroblasts and macrophage markers. It has been reported that macrophages are directly converted to fibroblast-like cells in the granulation tissue^11^. These results suggest that Cluster 2 is involved in the process of differentiation from macrophages to fibroblast-like cells. The fibroblast-like cells derived from the circulation included CD45-positive and CD45-negative cells (Fig. 5). As scRNA-seq demonstrates increased sensitivity than histological analyses, these data suggest that the process of fate conversion is active in such cells.

Cluster 2 cells do not express extracellular matrix genes except for vimentin; therefore, we suggest that these are not mature fibroblasts. As they express chemokine genes, such as CCL6, CCL9, CCL24, and CXCL16, they retain the features of macrophages and play roles in immune regulation.

The proposed fibrocytes, which are bone marrow-derived circulating cells, exhibit the characteristics of both fibroblasts and monocytes^13^. Fibrocytes usually migrate to damaged sites and contribute to their repair^13^. Although we could not obtain direct evidence to support that Cluster 2 originated from fibrocytes, our results indicated the possibility of this phenomenon. Further investigations are warranted to address this issue.

Taken together, our results support the assumption that circulating hematopoietic cells, most likely macrophages, migrate to and expand the activated omentum by changing their fate to fibroblast-like cells during inflammation. As Cluster 2 expresses various chemokines, they possibly play a role in recruiting and activating immune cells. Our results provide important information on the activation of the omentum and its application in regenerative medicine.

## Methods

### Mice

Mice were bred and maintained at the Kansai Medical University Research Animal Facility in accordance with the Kansai Medical University guidelines. Eight-week-old C57BL/6J mice were purchased from Shimizu Laboratory Supplies (Kyoto, Japan). *WT1*^*CreERT2/*+^ (JAX 010912) and *Rosa26*^*loxp-stop-loxpYFP/*+^ (JAX 006148) mice were purchased from Jackson Laboratories. *Rosa26*^*EGFP/*+^ and *Rosa26*^*ECFP/*+^ mice were provided by I. L. Weissman (Stanford University School of Medicine, USA)^6^. The experiments were approved in advance by the Kansai Medical University Welfare Committee. Tamoxifen (Sigma, St. Louis, MO, USA, T5648) was dissolved in corn oil (Sigma, C8267) and injected intraperitoneally into 8-week-old adult mice at a concentration of 9 mg/40 g of body weight.

### Parabiotic surgery

Parabiotic surgery was performed following a previously described procedure^5,14^. Parabiotic mice were injected intraperitoneally with 1 mL Bio-gel P-60 polyacrylamide beads (Bio-Rad Laboratories, Hercules, CA, USA, 1504160). After 7 days, the mice were euthanized, and the omentum was harvested.

### Histological examination

The omenta were subjected to fixation overnight with 4% paraformaldehyde at 4 °C. The fixed tissues were embedded in paraffin for conducting hematoxylin and eosin, elastica van Gieson, Azan, and silver impregnation staining. Paraffin-embedded sections were sliced to 4 μm thickness. The fixed tissues were immersed overnight in 30% sucrose solution at 4 °C and embedded in optimal cutting temperature (OCT) compound for immunostaining. Furthermore, 8 μm-thick frozen sections were sliced and stained using the following primary antibodies: (a) rat anti-F4/80 (1:50; ATCC, Manassas, VA, USA); (b) rat anti-CD45 (1:100 or 1:500; BioLegend, San Diego, CA; 103102); (c) ER-TR7 (1:100; Novus Biologicals, Centennial, CO, USA; NB100-64932); (d) rabbit anti-perilipin A (1:5000; Sigma; P1998); and (e) rabbit anti-vimentin (1:100; Abcam, Cambridge, UK; ab92547). The primary antibodies were incubated with the specimens for 16 h at 4 °C. The specimens were incubated with the following secondary antibodies at room temperature for 1 h: (a) Alexa-488-conjugated goat anti-rat IgG (1:200); (b) Alexa-594-conjugated donkey anti-rabbit IgG (1:200 or 1:400); (c) Alexa-594-conjugated donkey anti-rat IgG (1:400); (d) Alexa-647-conjugated goat anti-rabbit IgG (1:400); and (e) Alexa-647-conjugated chicken anti-rat IgG (1:200). Finally, the sections were stained with Hoechst 33342 (Thermo Fisher Scientific, Waltham, MA, USA; H3570). The frozen sections were analyzed using the BX63 fluorescence microscope (Olympus Corporation, Tokyo, Japan) and the FV3000 confocal laser scanning microscope (Olympus).

### Preparation of single cells from the omentum

Omental cells were isolated according to previously described methods with slight modifications^4,12^. The omenta were minced and subjected to digestion using 0.4% collagenase (C0130; Sigma) for 30 min at 37 °C. Single-cell suspensions were filtered using a 70-μm cell strainer (Corning, Corning, NY, USA, 352350) to remove large aggregates, and were centrifuged at 250×g for 5 min at 4 °C. Single-cell suspensions from parabiotic *Rosa26*^*ECFP/*+^ mice paired with *Rosa26*^*EGFP/*+^ mice were subjected to washing steps and resuspended in 50 mL staining buffer (2% fetal bovine serum and 0.1% NaN_3_ in phosphate-buffered saline [PBS]).

### 3′-single cell RNA-seq

The cells were subjected to staining using the 7-AAD Viability Staining Solution (Invitrogen, 00-6993-50) and lineage markers, rat anti-mouse CD11b-PE (BD Pharmingen, 557397), rat anti-mouse CD31-PE (BD Pharmingen, 553373), rat anti-mouse CD45-PE (BD Pharmingen, 553081), biotin rat anti-mouse TER-119 (BD Pharmingen, 553672), and streptavidin-PE (BD Pharmingen, 554061). The cells were then allowed to pass through a 35-μm cell strainer (Corning, 352235). Single cells were sorted using SH800 (SONY, Tokyo, Japan) in 2% bovine serum albumin/PBS. Sorted cells were subjected to processing using the Chromium Controller (10x Genomics, Pleasanton, CA, USA), Chromium Next GEM Single Cell 3’ GEM, Library & Gel Bead Kit v3.1 (10x Genomics, PN-1000128), and MGIEasy Universal Library Conversion Kit (App-A) (MGI, Shenzhen, China) following the manufacturer’s instructions. The library was sequenced using DNBSEQ-G400 (MGI).

### Downstream analysis of 3′-single cell RNA-seq data

The scRNA-seq output data were processed using the Cell Ranger pipeline (10x Genomics, version 4.0.0) with the required mouse reference (mm10) datasets. Using the Seurat R package (version 3.1.5)^15^, data on cells that contained more than 6000 expressed genes or those that contained more than 5% mitochondrial genes were removed. Data on genes that were expressed in less than one cell were also removed, resulting in the obtainment of information on 18,476 genes and 2456 cells. The expression of each gene was subjected to normalization and data were log-transformed. To identify cell clusters, principal component (PC) analysis was performed using data on highly variable genes. The optimal number of PCs for each sample was determined using a combination of jackstraw and elbow methods. The first 40 PCs were used with the Louvain algorithm to generate cell clusters, and 10 clusters were obtained with a resolution of 0.5 (cluster 0 = 714 cells, cluster 1 = 693 cells, cluster 2 = 284 cells, cluster 3 = 237 cells, cluster 4 = 159 cells, cluster 5 = 147 cells, cluster 6 = 110 cells, cluster 7 = 60 cells, cluster 8 = 27 cells, and cluster 9 = 25 cells). Nonlinear dimensional reduction and visualization were performed using the UMAP algorithm and the PCs selected above. Additionally, marker genes for the individual clusters compared to all other cells were identified using the Seurat FindAllMarkers function with default parameters. Data on the top 100 marker genes of cluster 2 were used as inputs for analysis using Metascape^16^ (http://metascape.org) for gene annotation and analysis.

### Ethics statement

The experiments were approved in advance by the Kansai Medical University Animal Experiment Committee (Approval Nos.: 20-110, 20-128), in accordance with the Guidelines for Animal Experimentation, Kansai Medical University and the ARRIVE guidelines (https://arriveguidelines.org).

## Supplementary Information

Supplementary Information 1.

Supplementary Information 2.

## Data Availability

The datasets used and analyzed in the current study will be available from the corresponding author upon reasonable request.
