# Author Correction: Cellular basis of omentum activation and expansion revealed by single-cell RNA sequencing using a parabiosis model

**DOI:** 10.1038/s41598-021-04282-9

**Published:** 2021-12-21

**Authors:** Kazuhiko Ishigaki, Keiki Kumano, Kyohei Fujita, Hiroo Ueno

**Affiliations:** Department of Stem Cell Pathology, Kansai Medical University, 2-5-1 Shin-machi, Hirakata, Osaka 573-1010 Japan

Correction to: *Scientific Reports* https://doi.org/10.1038/s41598-021-93330-5, published online 06 July 2021

The Supplementary Information file published with this Article contained an error in the Table S1 format, where the table was incorrectly converted into PDF.

This error has now been corrected in the Supplementary Information file that accompanies the original Article.

