# Supplementary material for: Cellular basis of omentum activation and expansion revealed by single-cell RNA sequencing using a parabiosis model
Source: Sci Rep. 2021 Jul 6;11:13958. doi: 10.1038/s41598-021-93330-5 (PMC8260800; doi:10.1038/s41598-021-93330-5)
Supplement: Supplementary file 2 — Supplementary Information 2. [file 41598_2021_93330_MOESM2_ESM.pdf]

Supplementary information
Supplementary figure legends

Supplementary Table 1. Highly expressed top 100 genes in each cluster

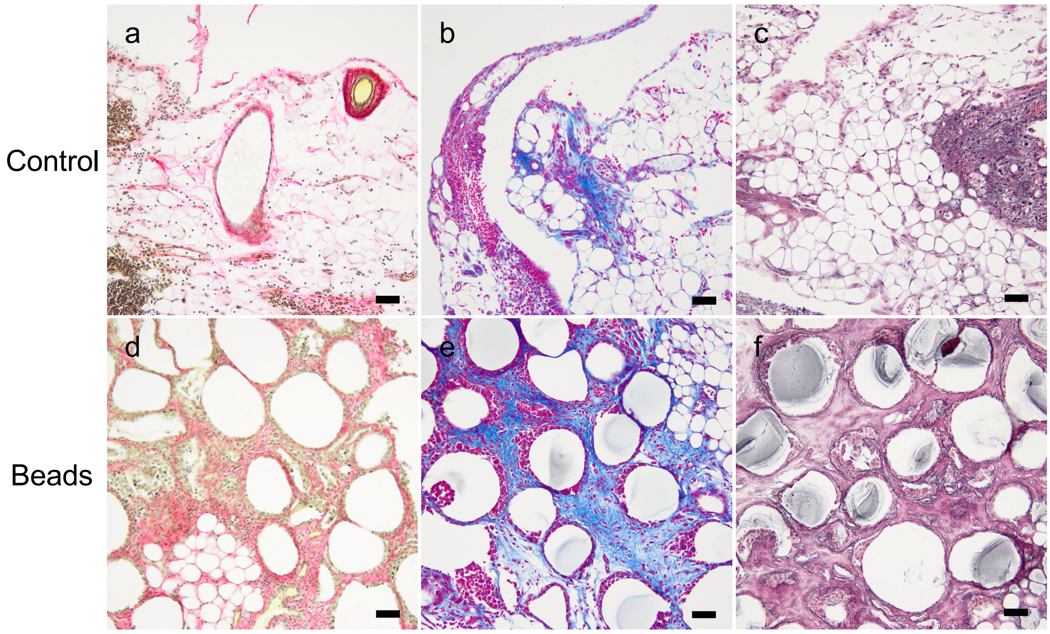

Supplementary Fig. 1. Representative images of the omenta of control and bead-injected mice

Immunohistochemistry of the omenta of C57BL/6J mice subjected to Elastica van Gieson (**a** and **d**), Azan (**b** and **e**), and silver impregnation (**c** and **f**) staining procedures. (**a**–**c**) Images of the tissue derived from the control mouse. (**d**–**f**) Images of the tissue derived from the bead-injected mouse. Bead-based treatment was conducted for a period of 7 days. Scale bars = 50 μm

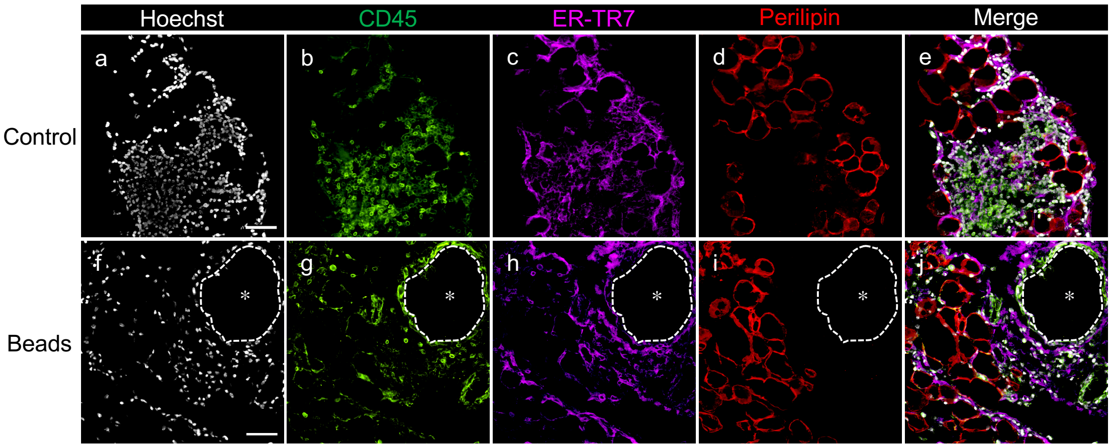

Supplementary Fig. 2. Representative images of the omenta of control and bead-injected mice

(a) Hoechst nuclear staining. **(b)** Expression pattern of CD45 in the same field as **a**. **(c)** Expression pattern of ER-TR7 in the same field as **a**. **(d)** Expression pattern of perilipin in the same field as **a**. (e) Merged image of **a**–**d**. **(f)** Hoechst nuclear staining. **(g)** Expression pattern of CD45 in the same field as **f**. **(h)** Expression pattern of ER-TR7 in the same field as **f**. **(i)** Expression pattern of perilipin in the same field as **f**. **(j)** Merged image of **f**–**j**. White dotted lines and asterisks (**f-j**) indicate polyacrylamide beads. Scale bars = 50 μm

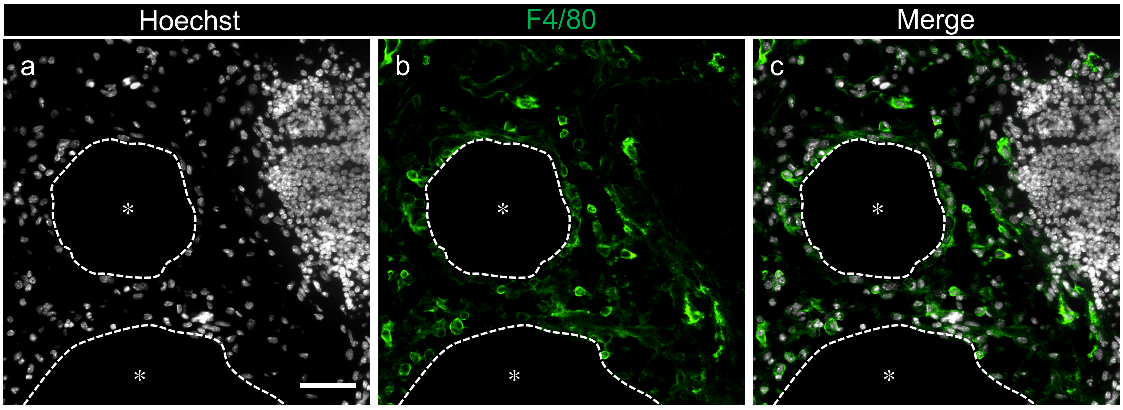

Supplementary Fig. 3. Representative images of the omenta of bead-injected mice
(a) Hoechst nuclear staining. (**b)** Expression pattern of F4/80 in the same field as **a**. (**c)** Merged image of **a** and **b**. White dotted lines and asterisks (**a**–**c**) indicate polyacrylamide beads. Scale bar = 50 µm

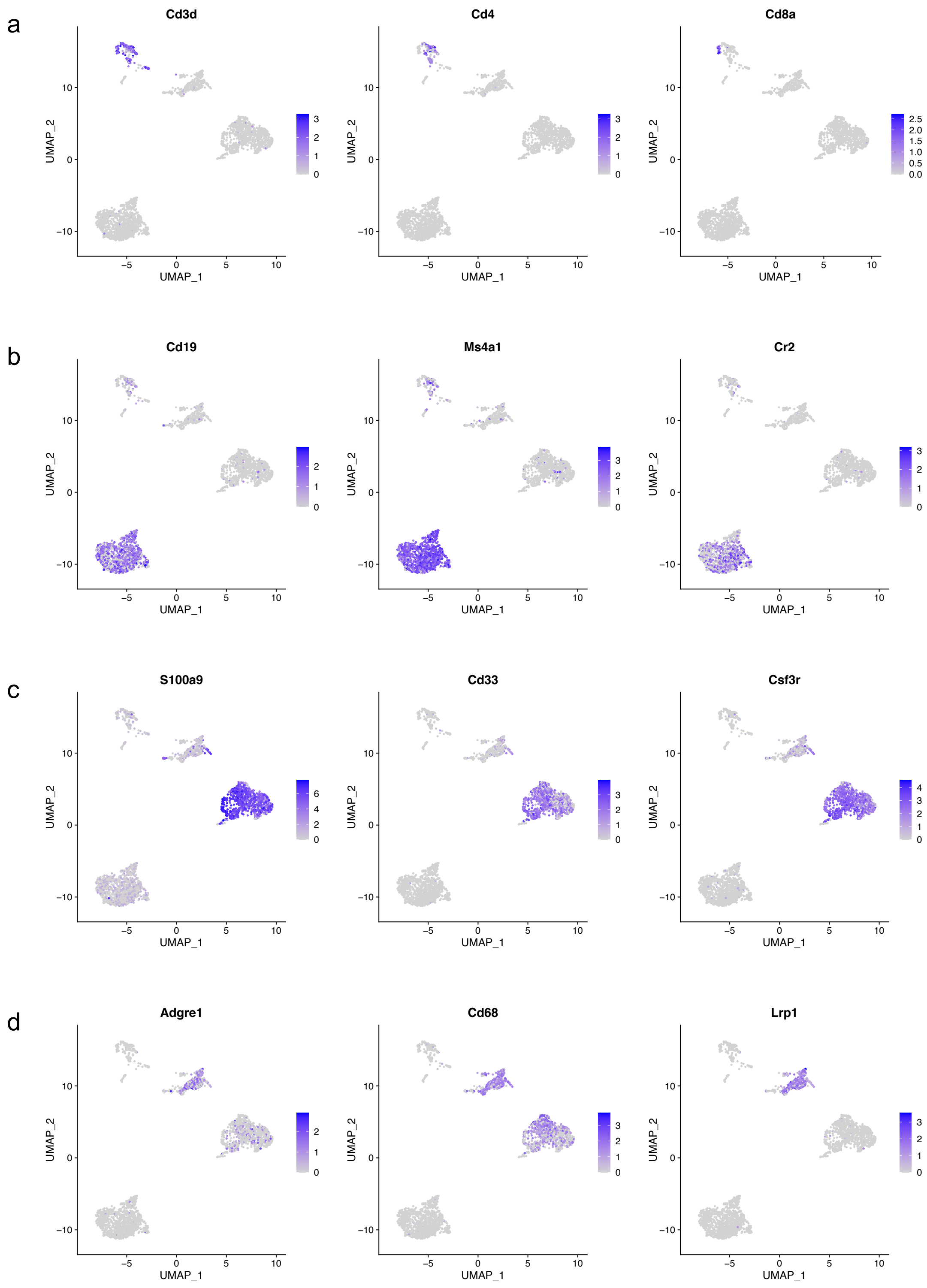

Supplementary Fig. 4. Characterization of clusters in the UMAP of scRNA-Seq using omentum cells (Fig. 4)

(a) Feature plots illustrating T cell markers *CD3d, CD4*, and *CD8a*. (**b)** Feature plots

illustrating B cell markers *CD19*, *Ms4a1* (also known as *CD20*), and *Cr2* (also known as *CD21*). (**c)** Feature plots illustrating myeloid cell markers *S100a9*, *CD33*, and *Csf3r*. (**d)** Feature plots illustrating macrophage markers *Adgre1* (also known as *F4/80*), *CD68*, and *Lrp1* (also known as *CD91*)).

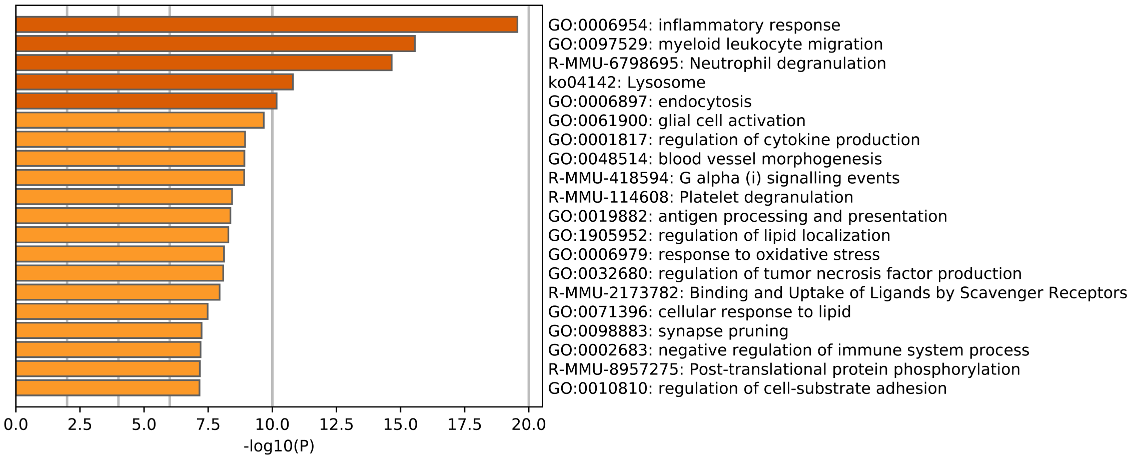

Supplementary Fig. 5. Enrichment analysis of the top 100 marker genes of Cluster 2 (based on Metascape analysis)

The top 20 gene ontology terms of Cluster 2. Data on the top 100 marker genes of Cluster 2 were used as inputs for analysis using Metascape. Enrichment analysis was performed using GO Biological Processes, Reactome Gene Sets, and KEGG Pathway. Heat map illustrating enriched terms colored according to *p*-values.

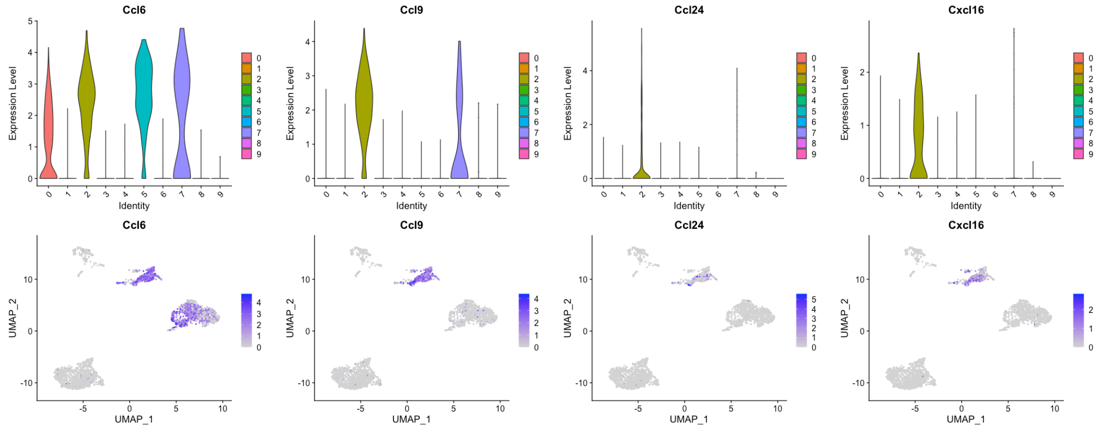

Supplementary Fig. 6. scRNA-Seq of omentum cells

Violin (top) and feature plots (bottom) illustrating chemokines *Ccl6*, *Ccl9*, *Ccl24*, and *Cxcl16*. These genes were identified as the specific markers of Cluster 2.

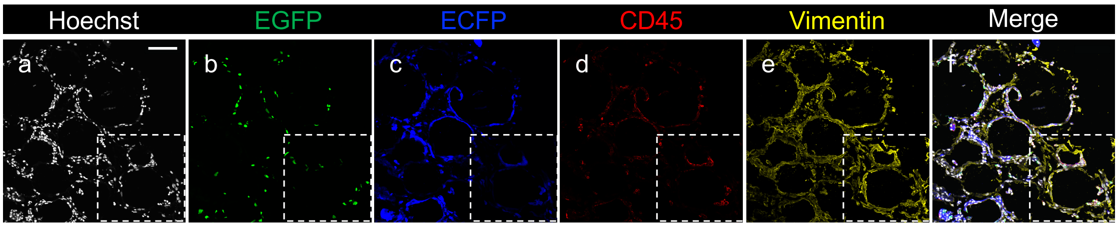

Supplementary Fig. 7. Representative confocal images of the omenta of parabiotic *Rosa26*$^{ECFP/+}$ mouse paired with *Rosa26*$^{EGFP/+}$ mouse

(**a**–**f**) Low-magnification images of **Fig. 5**. Dotted line squares in **a**–**f** show the area of the images in **Fig. 5**. Scale bar = 100 µm
